# Supplementary material for: Causal Relationship between Aging and Anorexia Nervosa: A White-Matter-Microstructure-Mediated Mendelian Randomization Analysis
Source: Biomedicines. 2024 Aug 16;12(8):1874. doi: 10.3390/biomedicines12081874 (PMC11351342; doi:10.3390/biomedicines12081874)
Supplement: Supplementary file 1 [file biomedicines-12-01874-s001.zip › Supplementary Figure/Figure S4/Singe SNP plot of MR analysis for TL to ubm-b-1490.pdf]

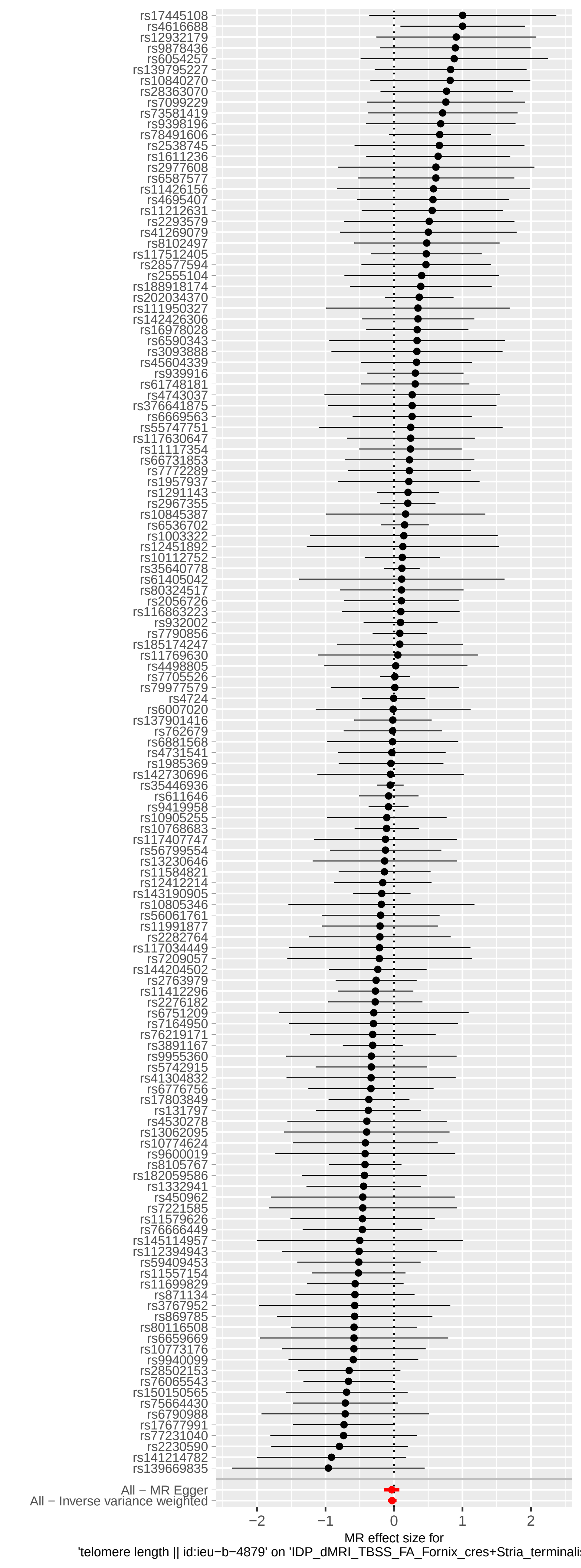
rs17445108
rs4616688
rs12932179
rs9878436
rs6054257
rs139795227
rs10840270
rs28363070
rs7099229
rs73581419
rs9398196
rs78491606
rs2538745
rs1611236
rs2977608
rs6587577
rs11426156
rs4695407
rs11212631
rs2293579
rs41269079
rs8102497
rs117512405
rs28577594
rs2555104
rs188918174
rs202034370
rs111950327
rs142426306
rs16978028
rs6590343
rs3093888
rs45604339
rs939916
rs61748181
rs4743037
rs376641875
rs6669563
rs55747751
rs117630647
rs11117354
rs66731853
rs7772289
rs1957937
rs1291143
rs2967355
rs10845387
rs6536702
rs1003322
rs12451892
rs10112752
rs35640778
rs61405042
rs80324517
rs2056726
rs116863223
rs932002
rs7790856
rs185174247
rs11769630
rs4498805
rs7705526
rs79977579
rs4724
rs6007020
rs137901416
rs762679
rs6881568
rs4731541
rs1985369
rs142730696
rs35446936
rs611646
rs9419958
rs10905255
rs10768683
rs117407747
rs56799554
rs13230646
rs11584821
rs12412214
rs143190905
rs10805346
rs56061761
rs11991877
rs2282764
rs117034449
rs7209057
rs144204502
rs2763979
rs11412296
rs2276182
rs6751209
rs7164950
rs76219171
rs3891167
rs9955360
rs5742915
rs41304832
rs6776756
rs17803849
rs131797
rs4530278
rs13062095
rs10774624
rs9600019
rs8105767
rs182059586
rs1332941
rs450962
rs7221585
rs11579626
rs76666449
rs145114957
rs112394943
rs59409453
rs11557154
rs11699829
rs871134
rs3767952
rs869785
rs80116508
rs6659669
rs10773176
rs9940099
rs28502153
rs76065543
rs150150565
rs75664430
rs6790988
rs17677991
rs77231040
rs2230590
rs141214782
rs139669835
All − MR Egger
All − Inverse variance weighted
−2
−1
0
1
2
MR effect size for
'telomere length || id:ieu−b−4879' on 'IDP_dMRI_TBSS_FA_Fornix_cres+Stria_terminali
